# Analyzing Cardiovascular Disease Research in the Arab Region: A Bibliometric Review From 2012 to 2022

**DOI:** 10.1155/2024/5915132

**Published:** 2024-10-12

**Authors:** Amjad Bani Hani, Alaa Tarazi, Yazan Hamadneh, Ahmad Al-Samahan, Rami Awad, Mohammad Kan'an, Mohammad Al-Kasaji, Nidal A. Younes

**Affiliations:** ^1^General Surgery Department, School of Medicine, The University of Jordan, Amman, Jordan; ^2^School of Medicine, The University of Jordan, Amman, Jordan

**Keywords:** Arab, bibliometric analysis, cardiology, cardiovascular diseases, productivity

## Abstract

**Background:** Cardiovascular diseases (CVDs) remain the leading cause of mortality, especially in low- and middle-income countries, many of which are in the Arab region. We aimed to conduct a bibliometric analysis to assess the scientific output concerning CVDs in the Arab region and the Middle East over the past decade.

**Methods:** A bibliometric search was conducted on PubMed and Scopus between 2012 and 2022. The study analyzed the number of publications, countries, institutional sources, authors, journals, and keywords. Visualization analysis was executed using various software tools, including R, VOSviewer, and CiteSpace.

**Results:** PubMed and Scopus yielded 5340 documents related to CVDs at the time of data collection. A total of 1263 documents were retrieved after screening based on specific inclusion criteria that guarantee the inclusion only for Arab region studies and authors. There has been a consistent increase in annual publications in recent years. The countries that contributed the most to research in this field were Egypt, Saudi Arabia, and the UAE. Although Egypt stood out as the most productive country, the institute with the highest number of documents was in Qatar. In addition, the Top 3 authors hailed from Qatar. Saudi Medical Journal leads the field as the most productive journal, followed closely by PLoS One and Angiology. Research topics of significant interest in the realm of CVDs include specific diseases such as heart failure, risk factors related to CVDs, genetic aspects of CVDs, and congenital disorders in infant's cardiac health.

**Conclusion:** This study provides a comprehensive overview of the current status of CVD research in the Arab region. It includes research hotspots that can guide authors in determining the necessary future investigations within this field. There is a clear call for further exploration of various aspects of CVDs in the Arab world. Strengthening cooperation between countries and institutes is needed.

## 1. Introduction

The Arab world (AW) is a group of 22 countries with similar language, culture, and religious backgrounds. These countries are home to more than 462,940,000 people, spanning across the Middle East and North Africa (MENA), and are part of the Arab League [1–3].

In 2019 alone, an estimated 18.6 million lives were lost to cardiovascular diseases (CVDs), constituting a significant 32% of global mortalities, making CVD the leading cause of death worldwide and a major cause of life-crippling morbidity [4–6]. Unfortunately, the majority of these deaths occur in low- and middle-income countries, particularly in regions such as countries in the MENA or sub-Saharan Africa [6].

The prevalence of CVDs and their associated risk factors in the Middle East reaches up to 10.1%. Moreover, the most common risk factors include dyslipidemia (43.3%), hypertension (26.2%), and diabetes (16%) [7], underlining the significance of these health issues in the region. Additionally, many Arab countries exhibit some of the world's highest rates of smoking among the adult population, ranging from 15.3% in Morocco to 53.9% in Lebanon, which is higher than the worldwide average [8], the prevalence of obesity [9], smoking, dyslipidemia, hypertension, diabetes, and other environmental risk factors such as poor air quality [10, 11], alongside the relatively low social development index (SDI) scores [12], genetic, and cultural factors [13]. These variables highlight the differences between the AW region and other regions in the world in addition to the pressing need for region-specific studies [14], as these studies can provide invaluable insights into the pathophysiology of CVDs in these populations.

This raises the compelling necessity for bibliometric studies scrutinizing published cardiovascular research in the Arab region. Bibliometric studies are defined as studies that focus on analyzing, measuring, and quantifying communication phenomena which include various aspects such as research articles, their authors, publication venues, keywords, citation counts, and many other criteria. Their primary aim is to build a formal and objective representation of a particular field, serving roles that are representative, explanatory, and administrative purposes [15]. Bibliometric studies have a wide range of applications extending to performance evaluation and benchmarking of institutes; selection of peer-reviewers, collaborators, and journals; analysis of research trends; guiding policymaking; and aiding scientists, institutes, funders, and governments in setting research priorities and allocating funds [16, 17], ultimately advancing the quality and impact of cardiovascular research in the region.

Multiple studies have examined CVD research output in the AW. These investigations have revealed a notable upward trend in CVD publications in recent years, although the region still lags behind the global output. These studies have taken into account the economic factors of each of the Arab countries and have compared their research output to that of countries such as the United States and the United Kingdom [18, 19].

Our study serves as a comprehensive bibliometric analysis encompassing all published CVD articles within the AW from 2012 to 2022. Its overarching aim is to provide a strategic map for shaping the future of research priorities in the Middle East region. This analysis evaluates the most pertinent scientific research about CVDs, with a keen focus on the research trends, influential authors, involved countries, prominent journals, and key contributing institutions.

## 2. Methods

### 2.1. Data Collection

Two databases were queried in this study, which are PubMed and Scopus. The search strategy was based on the following keywords: “heart or cardiology or interventional cardiology or cardiac or cardiovascular or vascular or blood vessels or myocardial infarction or acute coronary syndrome or angina or peripheral arterial disease or aortic disease or cardiovascular surgical procedures” and “Arab or authors with addresses in countries of the Arab league”. The timespan was set from January 1, 2012, to December 31, 2022. However, the query yielded a total of 5340 articles.

A comprehensive screening of all articles gathered from the two databases was conducted independently by two groups of three authors. Each group screened the title and the abstract of all the papers. Any discrepancies between the two groups were resolved through discussions between the groups.

The inclusion criteria for this study ensured that the selected articles were associated with the Arab region. These criteria encompassed articles where the first, senior, or corresponding author had an affiliation with an institution in one of the 22 Arab League nations. Additionally, the study had to be conducted within an Arab country in the Middle East. Furthermore, the selection process considered medical papers or reviews written in the English language or those with an available English translation. Importantly, only studies directly related to CVDs were included in the analysis.

Articles with first, corresponding, and senior authors who were not affiliated with an Arabic institute, unavailable English text, or irrelevant literature were excluded. The DOIs of screened articles from Scopus were compared to the articles from PubMed, and the duplicates were removed. Articles with no full text were also excluded from this study. In total, there were 1263 articles included in this study.

Gross domestic product (GDP), population, GDP per capita, research and development (R&D) expenditure (percent of GDP), total research expenditure, and expenditure per capita were extracted and calculated from the World Bank Databank and UNESCO Institute of Statistics (UIS) R&D SDG 9.5 dataset [20, 21, 22].

### 2.2. Data Analysis

CiteSpace 6.2.R2, Microsoft Excel 2019, VOSviewer, and R were used to perform bibliometric analysis.

CiteSpace is a citation visual analysis software developed from the background of Scientific metrics and knowledge visualization that is specifically used to identify potential knowledge contained in scientific literature [23]. It was utilized to visualize maps of cooperation between regions, countries, and institutions and analyze cooccurring words and authors' citations within specified timelines and keywords.

The parameters of CiteSpace were set as follows: timespan (2012–2022), years per slice (= 1), term source (title, abstract, author, keywords, and keywords plus), pruning (none), node type (chosen one at a time), and selection criteria (top 100% objects). Other parameters followed the default settings.

VOSviewer is another bibliometric software that is also used for constructing and viewing bibliometric maps, displaying them in certain types of clusters and various density colors [24]. In this bibliometric study, VOSviewer was used to visualize a map of cooccurrence keywords; it was also used to make a map of institutions. However, the links in VOSviewer indicate cooccurrence, and the thickness of the links depends on the strength values; that is, thicker lines indicate a stronger link [25].

Titles, authors, affiliations, keywords, and other parameters were extracted from the screened data in R [26] with the easyPubmed package [27]. The extracted data was cleaned in R using the stringer package [28].

Microsoft Excel was used to create a trend chart of annual publications and present the number of publications of each country, institution, and author. Also, it was used to generate a statistical chart of the publications and show the most used keywords, countries, institutions, and authors involved in our article.

Moreover, Microsoft Excel was utilized for screening and presenting information about countries, institutions, authors, and journals in an organized manner.

The *m* quotient or *m* index, which is a variation of the *h* index that takes into account the academic age of the author [29], was calculated for top authors.

## 3. Results

### 3.1. General Description of Data

**Figure 1:**
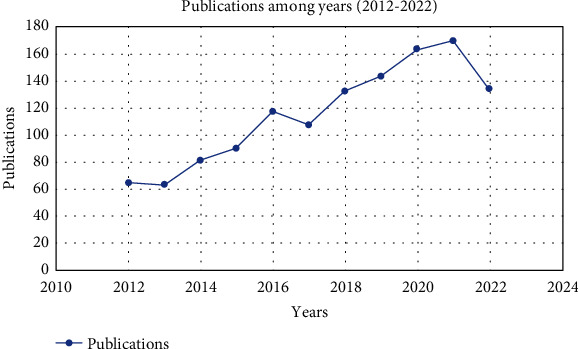
Number of publications in the Middle East from 2012 to 2022.

Over the past decade, there has been a notable surge in the publication of research papers in the field of CVDs within the Middle East. The quantity of publications within each year serves as a valuable indicator of the pace of research advancement during this period. As evident, between 2012 and 2022, the global annual publication counts experienced substantial growth, with the number of publications escalating from 64 publications in 2012 to 164 publications in 2022, as shown in Figure 1.

### 3.2. Distribution of Countries/Regions

**Figure 2:**
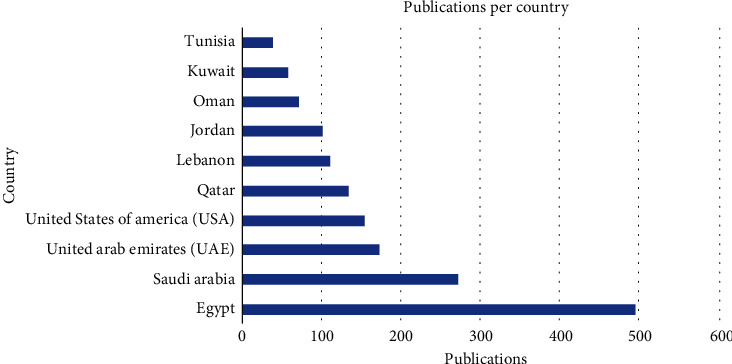
Number of publications per country in the studies of CVD in the Middle East.

A total of 1263 publications on CVDs were published by various Middle Eastern countries and regions in the last decade. The highest number of publications originated from Egypt, with 495 publications, accounting for 39.2% of the total. Following Egypt, Saudi Arabia contributed 271 publications (21.5%) and the United Arab Emirates with 172 publications (13.6%), as shown in Table 1 and Figure 2. These three countries accounted for more than half of all publications, underscoring their significant role in CVD research in the Middle East. However, the United States also made a substantial contribution, with 153 publications (12.1% of the total). Additionally, Egypt, Saudi Arabia, the UAE, and Qatar all spent more than a billion USD on R&D in 2022 which has been reflected in their research output. Additional information about the GDP, population, and research spending is in Table S1.

**Figure 3:**
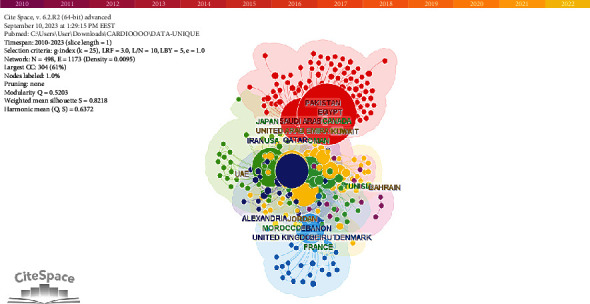
Visualization map of countries/regions in the studies of CVD in the Middle East.

The visualization map of the countries was generated by CiteSpace. As shown in Figure 3, each circular node on the map represents a specific country or region, with the size of each circle corresponding to the number of publications produced by that country/region. The lines connecting these circles represent a collaboration between countries/regions, and the width of these lines reflects the degree of cooperation, with wider lines indicating closer cooperation. The map illustrates several collaborations, particularly active among countries like Egypt, Saudi Arabia, and Qatar within the Middle East. Additionally, the presence of foreign countries on the map signifies their collaboration with Middle Eastern countries, underscoring the international nature of research partnerships in the field.

### 3.3. Distribution of Institutions

In the Middle East, Hamad Medical Corporation in Qatar is the leading institute for research publications in the field of CVDs. They have contributed 51 publications, as indicated in Table 2. Following closely is King Saud University in Saudi Arabia, with 41 publications, Royal Hospital in Oman with 26 publications, and Ain Shams University in Egypt with 24 publications. Notably, while Qatar has the highest number of publications from a single institute, Saudi Arabia has two institutes ranked among the Top 10, collectively accounting for 60 documents.

### 3.4. Distribution of Authors

A total of 5870 authors contributed to the literature on CVD publications in the Middle East, as presented in Table 3. Jassim Al Suwaidi, affiliated with the Department of Cardiology at Hamad Medical Corporation in Qatar, stands out as the most published author, with 38 documents representing 3.0% of the total. Followed closely by Rajvir Singh (33 documents) and Ayman El-Menyar (26 documents), both also affiliated with Qatar. Khalid F. Alhabib from Saudi Arabia follows, with 24 documents, and the list continues. Khalid F. Alhabib is the rising star of the list, as he is the youngest author on the list with the highest *h* index of 51 and the highest *m* index of 5.67.

Although Egypt had the highest number of articles as a country, the authors with the most articles predominantly come from Qatar.

**Figure 4:**
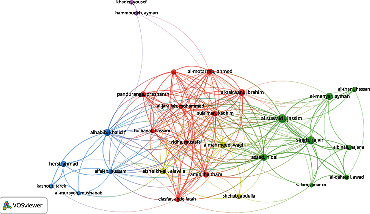
Visualization map of authors in the studies of CVDs in the Middle East.

In Figure 4, VOSviewer was used to create a visualization map that represents authors as nodes, with larger nodes indicating authors with a higher number of published articles. The thickness of lines connecting nodes indicates the extent of collaboration between authors. The map highlights a collaborative network among various authors in the field of CVDs. Notably, Jassim Al Suwaidi, the author with the highest number of articles, has engaged in numerous collaborations including a partnership with Rajvir Singh, Ayman El-Menyar, and Nidal Asaad, all of whom are affiliated with Hamad Medical Corporation. This collaborative network showcases the synergistic efforts among authors to advance research in this area.

### 3.5. Research Hotspot Analysis

**Figure 5:**
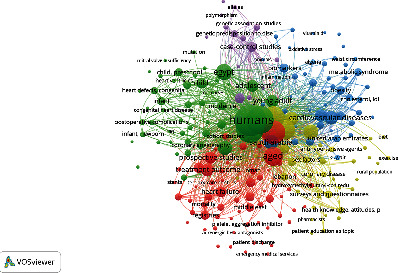
Cluster analysis of keywords associated with CVD research in the Middle East.

Keywords serve as the essence of a research paper, encapsulating the core topics of study.  Table 4 shows the high-frequency keywords. Humans (1010), female (790), and male (784) emerge as the Top 3 most frequently used keywords; this underscores the increasing focus on studies examining disparities related to CVDs between genders. Moreover, among these keywords, terms related to specific cardiac diseases such as “hypertension” and “heart failure” also rank among the Top 20 keywords. VOSviewer software was used to cluster the keywords. Each cluster is identified by a distinct color, indicating different research areas or hotspots.  Figure 5 reveals the presence of five prominent research clusters which are red, yellow, blue, purple, and green. Red clusters were composed of heart failure, stroke, and atrial fibrillation. The keywords of the yellow cluster were coronary disease, lifestyle, and smoking. The keywords of the blue cluster were atherosclerosis, metabolic syndrome, and cholesterol. While for the purple cluster, the keywords included alleles, mutation, and polymorphism. Finally, the green cluster keywords were composed of infant, heart ventricles, and cardiac surgical procedures. These keyword clusters highlight five distinct research directions or hotspots within the field of CVDs, ranging from gender disparities and risk factors to genetics, physiology, and clinical interventions. Understanding these clusters can provide valuable insights into the diverse aspects of cardiovascular research in the region.

### 3.6. Distribution of Journals

A total of 1263 articles on CVDs in the Middle East were published in 486 different journals. The Top 10 journals accounted for 247 articles, making up 19.6% of the total literature. The Saudi Medical Journal (42, 3.3%) had the highest number of publications in the field of CVDs, followed by PLoS One (33, 2.6%) and Angiology (28, 2.2%). The Top 10 journals were classified into two categories: JCR (Journal Citation Report) or Scopus, because half of these top journals were found in JCR with impact factor and quartile rankings; the remaining half were not listed in JCR but were available in Scopus, along with their respective quartiles. Among the Top 10 journals, PLoS One and Angiology had the highest impact factor. Five of the Top 10 included journals were present in JCR, indicating their recognized impact and quality. However, four of the top journals were not listed in JCR and were instead found in Scopus, as shown in Table 5, providing valuable research contributions in CVDs. It is also noteworthy to mention that Heart Views Journal did not have any classifications in either JCR or Scopus.

## 4. Discussion

### 4.1. General Information

Analysis of publications from the AW revealed a notable upward trend over the past decade ending, with 164 publications in 2022, indicative of growing interest in CVD research within the region. These findings align with a prior study that reported a surge in CVD-related publications across the AW from 2002 to 2015 [19]. It is particularly notable that the years coinciding with the COVID-19 pandemic witnessed a substantial increase in research output. This surge can be primarily attributed to the pandemic's profound impact on the cardiovascular system [30], prompting a surge in studies aimed at elucidating the intricate relationship between COVID-19 and CVDs. However, despite these encouraging trends, the AW still lags significantly behind other global regions in terms of CVD research output [18, 31].

The AW has a dynamic, political, and economic landscape, with many events occurring in the last 10 years relating to specific countries or the region as a whole. These events include the implementation of national initiatives such as Saudi Vision 2030 and the building of new universities and research institutes across the region [32]. Additionally, regional factors play an important role in shaping the current Arab landscape such as reduced economic income levels and economic turmoil like the Lebanese economic crisis [33], conflicts including the Iraqi conflict which had been ongoing since 2003, political instabilities, and conflicts following the Arab Spring such as Egypt, Syria, Yemen, and Libya [34, 35]. These factors have caused a shortage in the limited funding opportunities, further constraining research initiatives. Moreover, the region was affected by global events, most notably the SARS-COVID-19 pandemic [36]. An interesting additional factor is a historical emphasis on healthcare delivery rather than clinical research in medical schools and hospitals in the Arab region [32, 37].

These factors, combined with limited funding opportunities, a shortage of adequate research infrastructure, and qualified laboratory staff, have hindered progress in this field [18, 34].

Our findings have revealed that Egypt and Saudi Arabia stand out as the most productive countries in the field of CVD research, aligning with a previous study covering the years 2002–2015 [19]. Another bibliometric study on Behcet's disease articles among Arab countries also identified Egypt as one of the Top 5 most productive countries in this area, highlighting Egypt's research productivity [38]. Within this group of top countries, which includes Saudi Arabia, Qatar, and the United Arab Emirates—members of the Gulf Cooperation Council (GCC)—these countries prominently feature among the Top 5 in terms of productivity in cardiovascular research. This prominence can be largely attributed to their high-income status and high investment in research—especially in the last few years—compared to other Arab nations in the Middle East, as well as recent investments in medical research infrastructure, the establishment of new institutions, and programs, and strengthened commitment to scholarly pursuits [39]. Qatar's development of an academic health science system [40] has likely played a significant role in increasing its CVD publications. For Saudi Arabia, earlier research activity and increased research expenditure in recent years have contributed to its strong performance, while increased funding and the expansion of research institutes in the UAE have led to a notable surge in research output [41]. It is worth noting that the United States, while not strictly meeting the Middle East or Arab inclusion criteria in the articles considered, makes a substantial contribution, underscoring its significant collaborative role in cardiovascular research with Middle Eastern countries. Hamad Medical Corporation in Qatar boasts the highest output of CVD publications in the Middle East. This achievement aligns with Qatar's reputation as one of the leading countries in terms of research output. It is worth noting that three of the Top 10 most productive authors in the Arab region in the field of CVDs, namely, Al Suwaidi, Singh, and Asaad, are associated with the Hamad Medical Corporation. This underscores the significant impact of this institute and the quality of researchers it produces. The fact that Hamad Medical Corporation has the highest number of publications aligns with the presence of the most prolific authors, as shown in Table 3, being affiliated with this institution in Qatar.

On the other hand, Egypt is distinguished as the most prolific country in CVD research, with three leading institutions: Ain Shams University, Zagazig University, and the National Heart Institution. Collectively, these institutions contribute to 4.5% of the total CVD research output among various Arab institutions. This indicates Egypt's position as the most productive country, supported by a diverse range of research organizations, as demonstrated in another study [41]. However, considering the number of publications per GDP and population, there is significant room for improvement in Egypt.

The study found that the Saudi Medical Journal exhibited the most productive research output on CVDs. Remarkably, this journal was also identified as the most research-productive journal among Arab countries in a study done by El Rassi et al. [42]. Following closely behind were PLoS One and Angiology, which emerged as the most prolific journals in the cardiac field in the region, confirming the alignment of our results with the prevailing research focus. However, our analysis of journals indicated a notable trend. Researchers in the Middle East primarily submitted their papers to journals with relatively low impact factors. Only five of the Top 10 journals identified in our study were listed in the JCR for 2022. Among these, four had quartile rankings of Q3 or Q4 and impact factors of less than four, while the remaining five journals in the Top 10 were not even found in the JCR list, with four of them being presented in Scopus but with low quartile rankings. Notably, one journal, “Heart Views,” was not listed on JCR. This suggests a need for further evaluation in the field of cardiovascular research and a push from institutes and researchers to participate and conduct higher-quality research, which is an essential metric for the improvement of cardiovascular research in the Arab region.

### 4.2. The Hotspots and Frontiers

The identification of research frontiers and future directions within a specific research field heavily depends on the presentation and distribution of keywords and hotspots. The current research hotspots in CVDs in the Middle East can be identified through keyword clustering and the Top 20 keywords along with their total link strength, which are grouped into five different colored clusters.

The red cluster includes interconnected keywords such as heart failure (HF), atrial fibrillation, hospitalization, and acute coronary syndrome. These terms are closely related, as atrial fibrillation is often associated with acute coronary syndrome, which in turn can lead to HF. It is important to note that acute coronary syndrome is categorized as a type of coronary artery disease, which is a primary cause of HF in the Arab region [43]. Furthermore, this cluster encompasses the keywords COVID-19. HF is a common condition that can manifest at various stages during a COVID-19 infection. A multicenter study conducted in Italy revealed that acute HF can occur in COVID-19 patients during their hospitalization, and some of these patients had a history of preexisting HF, while others experienced new-onset HF despite no prior history of the condition [44].

The yellow cluster encompasses keywords such as coronary heart disease, smoking, awareness, and risk factors. This cluster suggests that studies conducted in the Middle East have explored various factors that may contribute to the development of heart diseases, including the impact of smoking, exercise habits, and dietary choices. A recent study conducted in the MENA highlighted these behavioral factors. It identified dietary risks, tobacco use, and low physical activity as the most significant contributing factors to coronary heart disease in the region [45]. The blue cluster focuses on keywords related to endocrine-related cardiovascular risk factors, including atherosclerosis, hypertension, diabetes mellitus, obesity, and metabolic syndrome. It is important to note that individuals in the Middle East bear a significant burden of hypertension and diabetes mellitus, both of which are well-known contributors to the heightened risk of various CVDs, potentially leading to conditions like ischemic stroke, and in severe cases, fatalities. A study by Verma et al., conducted in the Middle East and Africa to assess the prevalence of vascular risk among people with Type 2 diabetes (T2D), uncovered concerning findings. It revealed that one in five individuals with T2D in these regions had established atherosclerosis CVD, and 99.3% of them met high/very high-risk criteria set by the European Society of Cardiology [46]. These statistics indicate the profound impact these diseases have on cardiovascular health in the region and emphasize the urgent need for interventions aimed at reducing the risk factors associated with exacerbating cardiac conditions.

The purple cluster comprises keywords such as alleles, polymorphism, and myocardial infarction. This suggests a focus on genetic risk factors for CVDs among Arabs in the Middle East. A multicenter study conducted in Jordan indicated the importance of family history as a risk factor. The study revealed that 39.4% of patients who underwent percutaneous coronary intervention had a family history of premature CVD [13]. This finding highlights the pressing need for the development of screening and prophylactic programs to detect and mitigate the risk in individuals with a family history of CVD, potentially reducing their chances of developing these conditions.

The green cluster focuses on keywords like adolescents, infants, congenital heart disease, mutations, and Egypt, signifying an emphasis on congenital heart defects among newborns and young adults in the Middle East. One of the driving factors behind this focus is the high prevalence of consanguineous in Arab countries, with reported rates ranging from 20% to 50% in the MENA [47, 48]. This elevated rate of consanguinity is a significant contributor to the occurrence of congenital diseases. For instance, in Egypt, the incidence of congenital heart diseases among children has been estimated to be 5–6 per 1000 live births [49]. This high incidence may be attributed in part to the substantial frequency of consanguineous marriages in Egypt, which stands at 35.3% [50]. These statistics shed light on the abundance of publications addressing congenital cardiac diseases in the Middle East, particularly in Egypt, where the prevalence of such conditions is notably high [51].

## 5. Limitations

This study is subject to several limitations. Firstly, there is a discrepancy in the visual information and charts generated by VOSviewer and CiteSpace when analyzing the same data, which raises concerns about the consistency of the analysis conducted using these two tools. Secondly, the exclusion of non-English introduces the potential for source bias in our findings. Lastly, the continuously evolving nature of databases could result in the omission of certain research hotspots and frontiers in this article. However, we maintain that this study offers valuable insights into the prevailing trends and the current state of cardiovascular research within the Arab region.

## 6. Conclusion

This study presents a comprehensive bibliometric analysis of CVD research in the Arab region, offering valuable insights for further exploration in this field. Egypt emerges as the most prolific country, with the Hamad Medical Corporation leading among institutions, and Jassim Al Suwaidi stands out as the most productive author. The Saudi Medical Journal is noted as the most productive journal. Identified research hotspots cover various topics, including risk factors, genetic profiles, cardiovascular-related diseases, and infant cardiovascular morbidities. The results provide a summary of the current status of cardiology research among Arab regions, suggesting the need for future projects and improved infrastructure to enhance research productivity and journal impact in order to align with the increased incidence of CVD locally and globally. In conclusion, fostering research collaborations among Arab countries and institutions, coupled with a stronger focus on developing necessary research infrastructure by providing better opportunities for cardiologists to come up with more qualitative and quantitative articles, is essential for advancing CVD research in the region.

## Figures and Tables

**Table 1:** Top 10 most productive countries/regions for CVD research in the Middle East.

**Rank**	**Country**	**Publications**	**Percentage (** **N**/1263**)**	**Spending (%)**
1	Egypt	495	39.2%	1.02
2	Saudi Arabia	271	21.5%	0.46
3	United Arab Emirates (UAE)	172	13.6%	1.5
4	United States	153	12.1%	3.46
5	Qatar	133	10.5%	0.68
6	Lebanon	110	8.7%	No data
7	Jordan	100	7.9%	0.7
8	Oman	71	5.6%	0.28
9	Kuwait	57	4.5%	0.08
10	Tunisia	38	3.0%	0.75

**Table 2:** Top 10 most productive institutions for CVD research in the Middle East.

**Rank**	**Institute**	**Country**	**Publications**	**Percentage (** **N**/1263**)**
1	Hamad Medical Corporation	Qatar	51	4.0%
2	King Saud University	Saudi Arabia	41	3.2%
3	Royal Hospital	Oman	26	2.1%
4	Ain Shams University	Egypt	24	1.9%
5	Cleveland Clinic	United Arab Emirates (UAE)	21	1.7%
6	Mohammed Bin Rashid University of Medicine and Health Sciences	United Arab Emirates (UAE)	19	1.5%
7	Prince Sultan Cardiac Center	Saudi Arabia	19	1.5%
8	Jordan University of Science and Technology	Jordan	18	1.4%
9	Zagazig University	Egypt	17	1.3%
10	National Heart Institute	Egypt	16	1.3%

**Table 3:** Top 10 most productive authors in CVD research in the Middle East.

**Rank**	**Authors**	**Documents**	**Country**	**h** ** index**	**Institute**	**m** ** quotient**
1	Jassim Al Suwaidi	38	Qatar	45	Hamad Medical Corporation	1.73
2	Rajvir Singh	33	Qatar	51	Hamad Medical Corporation	2.22
3	Ayman El-Menyar	26	Qatar	37	Weill Cornell Medicine	2.47
4	Khalid F. Alhabib	24	Saudi Arabia	51	King Saud University	5.67
5	Nidal Asaad	24	Qatar	21	Hamad Medical Corporation	1.24
6	Ibrahim Al-Zakwani	23	Oman	30	Sultan Qaboos University	1.88
7	Wael Al Mahmeed	23	United Arab Emirates	42	Cleveland Clinic Abu Dhabi	2.63
8	Ahmed Al-Motarreb	19	Yemen	24	Sana'a University	1.33
9	Alawi A AL Sheikh-Ali	19	United Arab Emirates	16	Mohammed Bin Rashid University of Medicine and Health Sciences	0.94
10	Prashanth Panduranga	18	Oman	17	Royal Hospital	1.21

**Table 4:** Top 20 keywords related to CVD research in the Middle East.

**Rank**	**Keyword**	**Occurrence**	**Total link strength**
1	Humans	1010	11,063
2	Female	790	9684
3	Male	784	9545
4	Middle aged	595	7682
5	Adult	504	6348
6	Risk factors	374	4953
7	Aged	357	4911
8	Egypt	270	2904
9	Saudi Arabia	226	2356
10	Cross-sectional studies	217	2750
11	Retrospective studies	203	2497
12	Prevalence	192	2512
13	Cardiovascular diseases	190	2107
14	Treatment outcome	164	2092
15	Hypertension	154	1727
16	Young adult	152	2054
17	Adolescent	149	1911
18	Prospective studies	149	1951
19	Heart failure	117	1246
20	Child	109	1219

**Table 5:** Top 10 most productive journals in CVD research in the Middle East.

**Rank**	**Source**	**Count (% of 1263)**	**JCR or Scopus**	**IF (2022)**	**Quartile in category**
1	Saudi Medical Journal	42 (3.3%)	JCR	1.422	Q4
2	PLoS One	33 (2.6%)	JCR	3.752	Q2
3	Angiology	28 (2.2%)	JCR	3.299	Q3
4	Journal of the Saudi Heart Association	26 (2.1%)	Scopus	NA	Q3
5	BMC Cardiovascular Disorders	22 (1.7%)	JCR	2.174	Q4
6	Sultan Qaboos University Medical Journal	21 (1.7%)	Scopus	NA	Q3
7	Heart Views	21 (1.7%)	Neither JCR nor Scopus	NA	NA
8	Annals Saudi Medicine	20 (1.6%)	Scopus	NA	Q3
9	International Journal of Cardiology	18 (1.4%)	Scopus	NA	Q1
10	Cardiology in the Young	16 (1.3%)	JCR	1.023	Q4

Abbreviation: NA = not available.

## Data Availability

The data that support the findings of this study are available from the corresponding author upon reasonable request.
