# Supplementary material for: Analyzing Cardiovascular Disease Research in the Arab Region: A Bibliometric Review From 2012 to 2022
Source: Int J Vasc Med. 2024 Oct 12;2024:5915132. doi: 10.1155/2024/5915132 (PMC11490352; doi:10.1155/2024/5915132)
Supplement: Supporting Information — Additional supporting information can be found online in the Supporting Information section. [file 5915132.f1.docx]

**Supplementary table**

**Supplementary table 1. Top 10 most productive countries/regions for CVD research in Middle East with additional information about GDP and research spending.**

| Rank | Country | Population (thousand) | GDP million USD | GDP/Capita | Spending % | Spending (million) | Research spending/ Capita |
| --- | --- | --- | --- | --- | --- | --- | --- |
| 1 | Egypt | 110,990 | 476,748 | 4295.41 | 1.02 | 4,863 | 43.81 |
| 2 | Saudi Arabia | 36,409 | 1,108,149 | 30436.13 | 0.46 | 5,097 | 140.01 |
| 3 | United Arab Emirates (UAE) | 9,441 | 507,535 | 53758.61 | 1.5 | 7,613 | 806.38 |
| 4 | United states of America (USA) | 333,288 | 25,462,700 | 76398.49 | 3.46 | 881,009 | 2643.39 |
| 5 | Qatar | 2,695 | 237,296 | 88050.46 | 0.68 | 1,614 | 598.74 |
| 6 | Lebanon | 5,490 | 23,132 | 4213.48 | No data | No data | No data |
| 7 | Jordan | 11,286 | 47,451 | 4204.41 | 0.7 | 332 | 29.43 |
| 8 | Oman | 4,576 | 114,667 | 25058.35 | 0.28 | 321 | 70.16 |
| 9 | Kuwait | 4,269 | 184,558 | 43232.14 | 0.08 | 148 | 34.59 |
| 10 | Tunisia | 12,356 | 46,665 | 3776.71 | 0.75 | 350 | 28.33 |
